# TGF-β: A novel predictor and target for anti-PD-1/PD-L1 therapy

**DOI:** 10.3389/fimmu.2022.1061394

**Published:** 2022-12-19

**Authors:** Ming Yi, Tianye Li, Mengke Niu, Yuze Wu, Zhenyu Zhao, Kongming Wu

**Affiliations:** ^1^ Department of Oncology, Tongji Hospital of Tongji Medical College, Huazhong University of Science and Technology, Wuhan, China; ^2^ Department of Breast Surgery, The First Affiliated Hospital, College of Medicine, Zhejiang University, Hangzhou, China; ^3^ Department of Gynecology, The Second Affiliated Hospital of Zhejiang University School of Medicine, Hangzhou, China; ^4^ Department of Urology, Institute of Urology, Tongji Hospital, Tongji Medical College, Huazhong University of Science and Technology, Wuhan, China

**Keywords:** cancer biotherapy, cancer immunotherapy, tumor microenvironment, TGF-β, PD-1, PD-L1, bispecific antibody

## Abstract

Transforming growth factor-β (TGF-β) signaling regulates multiple physiological processes, such as cell proliferation, differentiation, immune homeostasis, and wound healing. Besides, TGF-β plays a vital role in diseases, including cancer. Accumulating evidence indicates that TGF-β controls the composition and behavior of immune components in the tumor microenvironment (TME). Advanced cancers leverage TGF-β to reshape the TME and escape immune surveillance. TGF-β-mediated immune evasion is an unfavorable factor for cancer immunotherapy, especially immune checkpoint inhibitors (ICI). Numerous preclinical and clinical studies have demonstrated that hyperactive TGF-β signaling is closely associated with ICI resistance. It has been validated that TGF-β blockade synergizes with ICI and overcomes treatment resistance. TGF-β-targeted therapies, including trap and bispecific antibodies, have shown immense potential for cancer immunotherapy. In this review, we summarized the predictive value of TGF-β signaling and the prospects of TGF-β-targeted therapies for cancer immunotherapy.

## 1 Background

**Figure 1:**
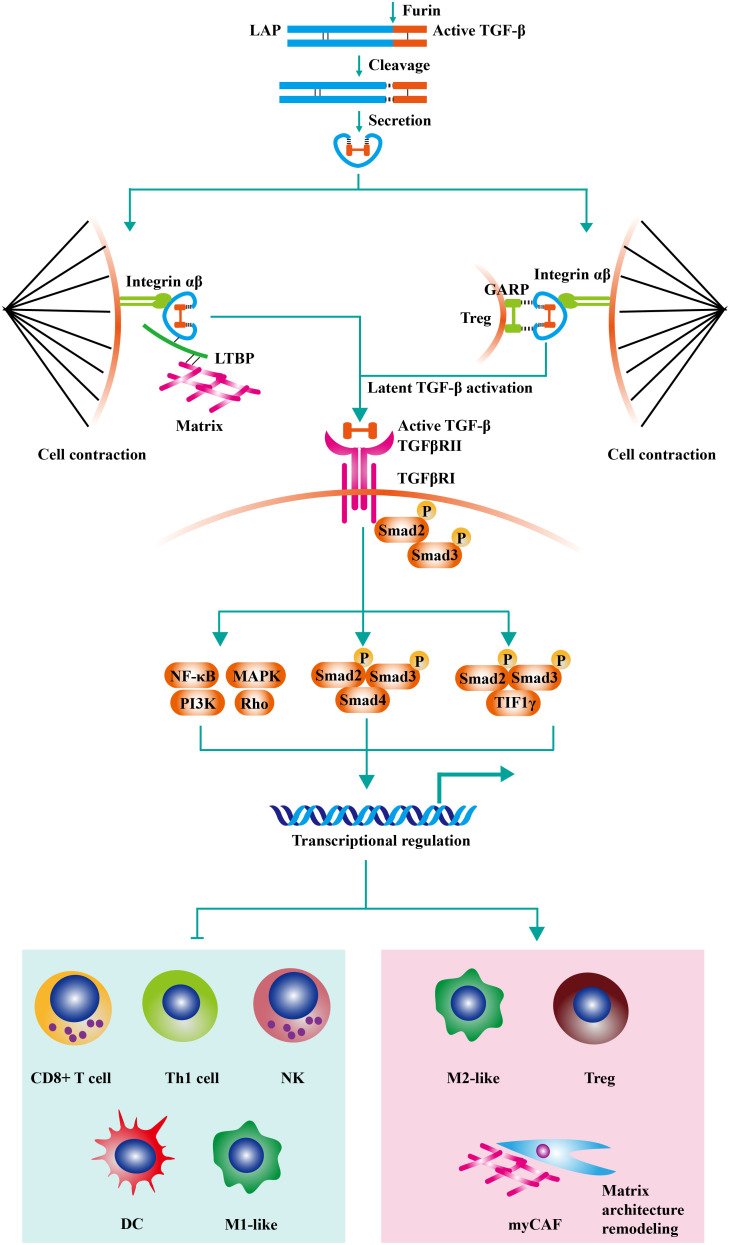
The negative effects of TGF-β signaling on anti-tumor immunity. Pro-TGF-β contains a long signal sequence, a long N-terminal sequence named latency-associated peptide (LAP), and a short C-terminal, which is the mature cytokine. Then, dimerized pro-TGF-β is cleaved by Furin (a protease) in Golgi complex. As a result, the bioactive TGF-β moieties are linked with LAP homodimer through disulfide bonds. The LAP encircles bioactive TGF-β moiety and hampers the binding of TGF-β with its receptor. After secretion, The LAP homodimer could anchor to Glycoprotein A repetitions predominant (GARP) on Treg or crosslink with extracellular matrix by latent TGF-β binding proteins (LTBPs). Then, active TGF-β is released by integrin-transmitted forces when cell contraction. TGF-β signaling is triggered by the interaction of TGF-β ligands with TGF-β type II receptors (TGFβRII). Following the recruitment and phosphorylation of TGF-β type I receptors (TGFβRI) by TGFβRII, SMAD2 and SMAD3 are phosphorylated and further assembled into trimeric complexes with SMAD4. The SMAD complexes could translocate into cell nucleus and regulate the expression of TGF-β-targeted genes. TGF-β acts on various immune cells in the tumor microenvironment, inducing the generation of a suppressive immune microenvironment. On the one hand, TGF-β inhibits the cytotoxic activity of CD8^+^ T cells, CD4^+^ T cells, and NK cells. On the other hand, TGF-β increases the proportion of regulatory T cells (Treg) and M2-like macrophage. Moreover, recent studies have found that TGF-β modulates the activity of tumor-associated fibroblast (CAF) and increases the content of collagen fibers in the tumor stroma (contributed mainly by myCAF). The thickened collagen fibers surrounding the tumor tissue are detrimental to lymphocyte infiltration, resulting in an immune-excluded tumor type. Adapted from Bai et al, 2019 (3).

Transforming growth factor-β (TGF-β) exists in the extracellular matrix as latent precursors with prodomain, and the transformation from latent pro-TGF-β molecule to active TGF-β is a multiple-step process (1). Firstly, pro-TGF-β contains a long signal sequence, a long N-terminal sequence named latency-associated peptide (LAP), and a short C-terminal, which is the mature cytokine (2). Then, dimerized pro-TGF-β is cleaved by Furin (a protease) in Golgi complex. As a result, the bioactive TGF-β moieties are linked with LAP homodimer through disulfide bonds. The LAP encircles bioactive TGF-β moiety and hampers the binding of TGF-β with its receptor. After secretion, The LAP homodimer could anchor to Glycoprotein A repetitions predominant (GARP) on the cell surface or crosslink with the extracellular matrix by latent TGF-β binding proteins (LTBPs). Then, active TGF-β is released by integrin-transmitted forces when cell contraction **(** **Figure 1** **)** (4).

TGF-β signaling is triggered by the interaction of TGF-β ligands with TGF-β type II receptors (TGFβRII) (5). Following the recruitment and phosphorylation of TGF-β type I receptors (TGFβRI) by TGFβRII, SMAD2 and SMAD3 are phosphorylated and further assembled into trimeric complexes with SMAD4 (6). The SMAD complexes could translocate into cell nucleus and regulate the expression of TGF-β-targeted genes, including *TWIST1*, *SNAI1*, and *SNAI2* (7). Besides canonical SMAD signaling, TGF-β can initiate non-SMAD signalings, such as PI3K-AKT, MAPK, and RHO-ROCK pathways (8–10). TGF-β signaling plays a vital role in embryonic development and homeostasis by controlling cell proliferation, apoptosis, survival, differentiation, and stem-cell self-renewal (11).

TGF-β is a bifunctional cytokine in cancer, acting as tumor promoter and suppressor (12). For healthy cells and early-stage cancer cells, TGF-β inhibits tumorigenesis by inducing cell-cycle arrest (13). However, for late-stage cancers, cancer cells could bypass TGF-β-mediated apoptosis by mutating core components of TGF-β pathway (14). Contrarily, TGF-β promotes tumorigenesis by inducing epithelial-to-mesenchymal transition (EMT), eventually contributing to enhanced metastasis and chemoresistance (15–17). Besides, TGF-β also supports tumor progression by improving angiogenesis and immune evasion (4, 18). This transformation of TGF-β from tumor suppressor to tumor promoter is an important biological characteristic for advanced cancers (19).

The discovery of immune checkpoints and the development of drugs represented by programmed cell death protein 1/programmed cell death ligand 1 (PD-1/PD-L1) monoclonal antibodies are landmark events in cancer immunotherapy (20–24). Anti-PD-1/PD-L1 treatments have shown potent and sustained antitumor effects in patients across multiple cancer types (25–32). However, the low response rate is a crucial drawback of anti-PD-1/PD-L1 therapies, and ideal molecular markers are unavailable to select patients (33–35). The classical cancer-immunity cycle model describes antitumor immunity as a cascade of multistep cascade responses (36). PD-1/PD-L1 axis in the tumor is not the only immunosuppressive pathway (37). It has been shown that hyperactive TGF-β signaling in the tumor microenvironment (TME) can broadly modulate multiple immune cell activities, reshape the TME, and collectively participate in tumor cell immune escape (3). The TGF-β and PD-1/PD-L1 pathways are independent of and complementary to each other. Recent studies have shown that TGF-β is a determinant for anti-PD-1/PD-L1 therapies, which could effectively predict treatment efficacy (38–40). Therefore, constructing TGF-β-involved predictive biomarkers and exploring TGF-β-targeted therapies are valuable to cancer immunotherapy.

## 2 TGF-β signaling-targeted antitumor agents

**Figure 2:**
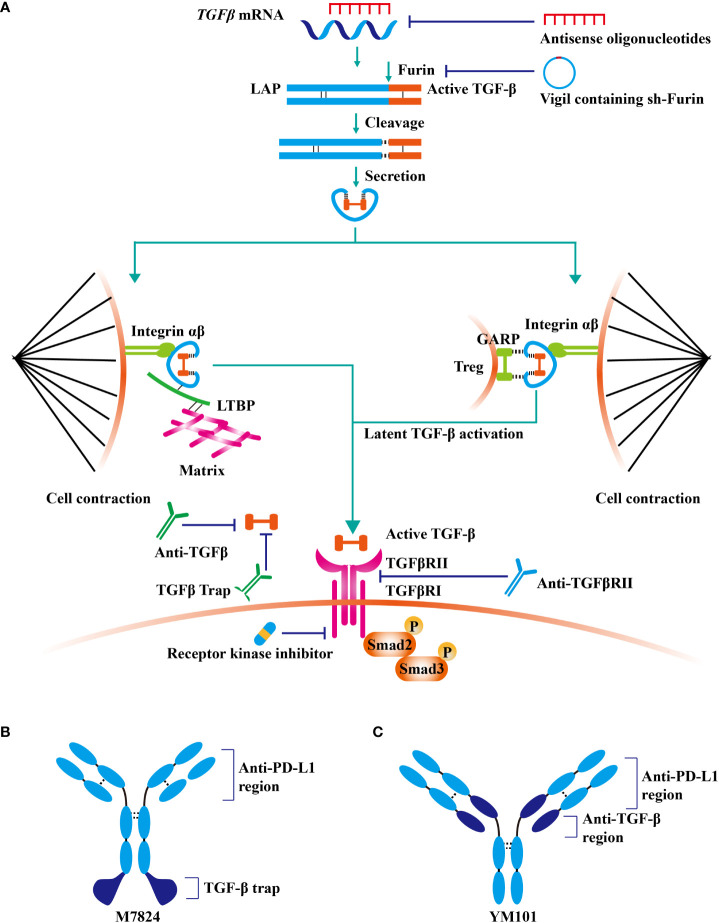
TGF-β signaling-targeted antitumor agents. At present, TGF-β signaling has been a hot therapeutic target for cancer investigators, and enormous efforts have been expended on the development of TGF-β-targeted agents. **(A)** TGF-β blockade strategies, including monoclonal antibodies (containing bispecific antibodies), ligand traps (containing bi-functional proteins), receptor kinase inhibitors, vaccines, and antisense oligonucleotides, are under clinical evaluation. **(B)** The structure of fusion protein M7824. **(C)** The structure of bispecific antibody YM101.

Given that TGF-β contributes to cancer immune evasion and immunotherapy resistance, blocking TGF-β could overcome immunotherapy resistance by reprogramming the TME. At present, TGF-β signaling has been a hot therapeutic target for cancer investigators, and enormous efforts have been expended on the development of TGF-β-targeted agents (41). TGF-β blockade strategies, including monoclonal antibodies (containing bispecific antibodies), ligand traps (containing bi-functional proteins), receptor kinase inhibitors, vaccines, and antisense oligonucleotides, are under clinical evaluation **(** **Table 1** **and** **Figure 2** **)** (42).

**Table 1:** Agents targeting TGF-β signaling pathway.

Classification	Agent	Target	Company/Authors
Antibody	Fresolimumab	TGF-β1/2	Genzyme
	SRK181	TGF-β1	Scholar Rock
	LY3022859	TGFβRII	Eli Lilly
	264RAD	Integrin αvβ6	AstraZeneca
	1D11	TGF-β1/2/3	Genzyme
	2G7	TGF-β1/2/3	Genentech
	YM101	TGF-β1/2/3 and PD-L1	YZY Biopharma
Receptor kinase inhibitor	Vactosertib	TGFβRI	MedPacto
	Galunisertib	TGFβRI	Eli Lilly
	LY3200882	TGFβRI	Eli Lilly
	LY573636	TGFβRI	Eli Lilly
	LY2109761	TGFβRI/II	Eli Lilly
	SB-431542	TGFβRI	GlaxoSmithKline
	SB-505124	TGFβRI	GlaxoSmithKline
	IN-1130	TGFβRI	In2Gen
Trap	AVID200	TGF-β1/3	Forbius
	Luspatercept	TGF-β1/2/3	Acceleron
	M7824	TGF-β1/2/3 and PD-L1	Merck KGaA
	SHR-1701	TGF-β1/2/3 and PD-L1	Hengrui
Antisense oligonucleotides	AP 12009	TGF-β2	Antisense Pharma
	AP 11014	TGF-β1	Antisense Pharma
Cancer vaccine	Vigil	TGF-β1/2	Gradalis
	Lucanix	TGF-β2	NovaRx

### 2.1 Antibodies targeting TGF-β or its receptor

Fresolimumab (also termed GC1008) is a pan-TGF-β blockade antibody developed by Genzyme for fibrotic diseases and cancers (43). Fresolimumab exhibited antitumor activity in renal cell carcinoma and melanoma with acceptable safety (43). Besides, in metastatic breast cancer, 10 mg/kg fresolimumab combined with irradiation outperformed 1 mg/kg fresolimumab plus irradiation in overall survival (Hazard ratio =2.73, *P* = 0.039) (44). The higher dose of fresolimumab was correlated with increased peripheral blood mononuclear cell and expanded CD8 memory T cell pool (44). Additionally, pan-TGF-β blockade antibodies 1D11 (developed by Genzyme) and 2G7 (developed by Genentech) exhibited antitumor activity in preclinical studies (45, 46). Notably, selective anti-TGF-β1 antibody SRK181 was sufficient to relieve the resistance to immune checkpoint inhibitors in murine models (47).

Y3022859 is an IgG1 antibody targeting TGFβRII (developed by Eli Lilly). In the phase 1 study of advanced solid tumors, the dose of more than 25 mg was unsafe in consideration of cytokine storm (48). Besides, anti-αvβ6 integrin antibody 264RAD (developed by AstraZeneca) could suppress TGF-β signaling by inhibiting latent TGF-β activation. The antitumor effect of 264RAD has been validated in multiple murine tumor models (49–51). Moreover, GARP, a protein mainly expressed on Treg surface, acts as the docking receptor to concentrate latent TGF-β (52). Selectively inhibiting GARP on Treg by antibody targeting GARP-TGF-β1 complexes effectively retarded tumor growth and relieved resistance to anti-PD-1/PD-L1 resistance (53). Notably, YM101 is an anti-PD-L1/TGF-β bispecific antibody (developed by Yi et al), which could simultaneously suppress PD-L1 and TGF-β signaling pathways (54). The preclinical data demonstrated YM101 effectively reprogrammed the TME and reserved immunotherapy resistance (54–56).

### 2.2 TGF-β receptor kinase inhibitor

TGF-β receptor kinase inhibitors block TGF-β signaling by occupying the ATP-binding domain of receptor (57). Vactosertib (developed by MedPacto) is a small-molecule inhibitor of TGFβRI (58). Vactosertib retarded tumor growth and prolonged survival in murine models by inhibiting EMT, cancer stemness, and metastasis (59–61). Also, galunisertib is a TGFβRI inhibitor developed by Eli Lilly (62, 63). Galunisertib showed potent antitumor activity in murine breast cancer, hepatocellular carcinoma, colon cancer, and lung cancer models (62). In clinical studies, galunisertib plus gemcitabine improved the overall survival of pancreatic cancer, relative to gemcitabine monotherapy (64). Besides, in the single-arm phase 2 trial of advanced rectal cancer, galunisertib combined with neoadjuvant chemoradiotherapy was tolerated, with an improved response rate (32%) (65). However, in the phase 2 study of recurrent glioblastoma, patients who received lomustine did not benefit from additional galunisertib treatment (66). Similarly, in a phase 1b study, galunisertib could not enhance the efficacy of ramucirumab in advanced hepatocellular carcinoma (67). LY573636 is a TGF-βRI inhibitor developed by Eli Lilly as well (68). Although several clinical trials showed that LY573636 had tolerable toxicity (69, 70), the results of the phase 2 study indicated that the autitumor effect of LY573636 was modest in NSCLC patients (71). At present, more than ten TGF-β receptor kinase inhibitors are in clinical or preclinical evaluations, including but not limited to LY2109761 (developed by Eli Lilly) (72), SB-431542 (developed by GlaxoSmithKline) (73), SB-505124 (developed by GlaxoSmithKline) (74), and IN-1130 (developed by In2Gen) (75, 76).

### 2.3 TGF-β trap

AVID200 (developed by Forbius/Bristol-Myers Squibb) is a computationally-designed trap that could effectively neutralize TGF-β1 and TGF-β3, with weak activity against TGF-β2 (77). The data of animal and human showed AVID200 enhanced antitumor immune response and reduced protumor and cardiotoxic effects caused by TGF-β2 blockade (77). Additionally, luspatercept (developed by Acceleron Pharma and Celgene) is a fusion protein containing the extracellular domain of human activin type 2B receptor and IgG, which has been approved as an erythroid maturation agent for β-thalassemia (78–80). Furthermore, soluble betaglycan (reported Bandyopadhyay et al.) inhibited angiogenesis, tumor growth, and metastasis in mice by antagonizing TGF-β (81).

M7824 (developed by Merck KGaA) is a bifunctional fusion protein consisting of anti-PD-L1 antibody and extracellular domain of the TGFβRII (82). M7824 showed potent antitumor activity in preclinical and phase 1 clinical studies by restoring antitumor immunity (82, 83). Similarly, anti-PD-L1/TGFβR fusion protein SHR-1701 (developed by Hengrui) overcame anti-PD-1/PD-L1 resistance in lung cancer (84).

### 2.4 Antisense oligonucleotides

Antisense oligonucleotides could directly silence genes participating in cancer progression. AP 12009 (developed by Antisense Pharma) is an antisense oligodeoxynucleotide targeting TGF-β2 (85). The data from phase IIb study of high-grade glioma demonstrated that 10 µM AP 12009 improved patients’ overall survival (86). Besides, other antisense oligonucleotides targeting TGF-β, such as AP 11014 and AP 15012, were still in preclinical tests (87, 88).

### 2.5 Cancer vaccine

Some cancer vaccines contain components suppressing TGF-β signaling pathway. Vigil (also termed gemogenovatucel-T, developed by Gradalis) is an autologous cancer vaccine that expresses granulocyte-macrophage colony-stimulating factor and decreases the expression of furin and its downstream TGF-β1 and TGF-β2 (89). In the phase 2b trial of advanced ovarian cancer, although vigil was well tolerated in patients, the primary endpoint was not met (90). Further investigations in other types of cancers are still undergoing (89). Moreover, Lucanix (also known as belagenpumatucel-L, developed by NovaRx) consists of allogeneic NSCLC cells transfected with the plasmid encoding TGF-β2 antisense gene (91, 92). In the phase III study NCT00676507, Lucanix improved the overall survival of NSCLC patients, especially these received prior chemotherapy or radiation (93).

## 3 Immune checkpoint inhibitor and its predictive biomarkers

PD-1/PD-L1 is an important signaling pathway to suppress immune responses and maintain autoimmune homeostasis (94, 95). However, in the TME, the hyperactive PD-1/PD-L1 pathway inhibits immune surveillance. It is traditionally believed that PD-L1, which is highly expressed on tumor cells, binds to PD-1 on the surface of T cells and suppresses the activity of T cells (96). PD-1/PD-L1 monoclonal antibody rescues T cells and restores antitumor immunity by blocking this negative immunomodulatory signal (97, 98). Recent studies have found that anti-PD-L1 antibodies also activate dendritic cells (DC) (99) and natural killer (NK) cells (100). Although PD-1/PD-L1 monoclonal antibodies are approved for the treatment of various cancers and have shown promising results in some patients, the problem of low objective response rates has not been effectively addressed (82, 101, 102). Therefore, screening for molecular biomarkers adapted to PD-1/PD-L1 therapy is an urgent issue at the present stage.

In terms of clinical efficacy, PD-L1 expression could not predict patient outcomes well, and even some patients whose tumors do not express PD-L1 can benefit from anti-PD-1/PD-L1 treatment (103–105). Apart from PD-L1 level, other predictive biomarkers have been identified, including tumor mutational burden (TMB) (106), mismatch repair (MMR) deficiency (107), the status of tumor-infiltrating lymphocyte (TIL) (108), immunosuppressive cell populations (109), oncogenic driver mutations (110–112), neoantigen repertoire (113), gut microbiota (114–116), inflammation-related genes (117, 118), extracellular vesicles (119), and patient’s clinical characteristics (120).

## 4 The role of TGF-β in cancer immunology and immunotherapy

High TGF-β in tumor tissues is mainly produced by tumor cells and mesenchymal cells. TGF-β promotes EMT of tumor cells and acts on various immune cells in the TME, inducing the generation of a suppressive immune microenvironment (121). On the one hand, TGF-β inhibits the cytotoxic activity of CD8^+^ T cells, CD4^+^ T cells, and NK cells. On the other hand, TGF-β increases the proportion of regulatory T cells (Treg) and myeloid-derived suppressor cells (MDSC) (122–125). Moreover, recent studies have found that TGF-β modulates the activity of tumor-associated fibroblast (CAF) and increases the content of collagen fibers in the tumor stroma (126). The thickened collagen fibers surrounding the tumor tissue are detrimental to lymphocyte infiltration, resulting in an immune-excluded tumor type (126). It is generally believed that this type of tumor does not respond to anti-PD-1/PD-L1 therapy, while antagonizing the TGF-β signaling pathway significantly improves anti-PD-1/PD-L1 therapeutic resistance and enhances the effect of antitumor immunotherapy (53, 127). Actually, although CAF was broadly classified into myofibroblastic (myCAF) and inflammatory and growth factor-enriched subgroups, some specific phenotypes are validated to participate in tumor progression as well (128). Besides, Grauel et al. found that TGF-β blockade induced the differentiation of IFN-licensed CAF, enhanced T cell recruitment and infiltration, and improved the effect of anti-PD-1 (129). Moreover, Krishnamurty identified a TGF-β-dependent CAF cluster with highly expressed LRRC15, which could support tumor progression by limiting T cell activity. Abrogating LRRC15^+^ CAF also significantly enhanced the efficacy of anti-PD-1 in mouse models (130).

Microsatellite-stable (MSS) colorectal cancer (CRC) is generally regarded as the cold tumor with poor immunogenicity and scare immune cell infiltration, which is unlikely to benefit from anti-PD-1/PD-L1 (131). However, this type of CRC could be conquered by the combination of anti-TGF-β and anti-PD-1/PD-L1 (132). Tauriello et al. established a metastatic CRC model by genetically engineering *Apc*, *Kras*, *Tgfbr2*, and *Trp53* quadruple mutant mice (132). Metastatic cancer tissues display characteristics of human MSS CRC: low mutation burden, T cell depletion, and TGF-β activation (132). Normal intestinal mucosa and adenoma had T cell infiltration in the mesenchyme, but not in adjacent cancer tissue (132). Anti-PD-1/PD-L1 treatment had limited effects on these tumors, while TGF-β inhibitors increased the sensitivity of anti-PD-1/PD-L1 treatment (132). Further investigations showed that combination therapy upregulated T-bet and IFN-γ levels in CD4^+^ Th1 cells and increased GZMB generation in CTLs, eventually eradicating metastases and prolonging survival (132). The results support that the TME with hyperactive TGF-β signaling caused T cell depletion and a decrease in Th1 effector cells, leading to cancer immune escape (132).

Besides, Mariathasan et al. analyzed cancer tissues from patients with metastatic urothelial carcinoma receiving anti-PD-L1 treatment (126). The responders were characterized by high PD-L1 expression, high tumor mutation burden/neoantigen, and CD8^+^ effector T cells (126). The non-responders had tumor tissue containing dense mesenchymal stroma, CAF with high TGF-β activity, and T cell deficiency (126). The mouse breast cancer EMT-6 model mimicked the phenotype of epithelial carcinoma, where blocking either PD-L1 or TGF-β alone was ineffective (126). Combined inhibition of TGF-β and PD-1 signaling reduces TGF-β activity in stromal cells, promotes T cell infiltration into the tumor, stimulates a robust immune response, and leads to tumor regression (126). In conclusion, several studies have shown that TGF-β pathway activity is hyperactivated in anti-PD-1/PD-L1-resistant tumor tissues (3). The high expression of TGF-β in the TME suppresses the antitumor immune response (3). The immunosuppressive mechanisms of TGF-β and PD-1/PD-L1 pathways on tumors are independent and complementary, promoting the escape from immune surveillance (36).

## 5 The predictive value of TGF-β signaling for anti-PD-1/PD-L1 treatment

In parallel with the immunosuppressive role of TGF-β in cancer immunology, the predictive value of TGF-β signaling in anti-PD-1/PD-L1 therapies has been well documented in multiple clinical studies. In the single-arm phase 2 study NCT02662309, 95 muscle-invasive urothelial cancer patients were recruited and received anti-PD-L1 treatment before cystectomy (38). In this study, the presence of preexisting activated CD8^+^ T cells (dual CD8 and GZMB positive staining) in the tumor was closely correlated with patient outcomes. Moreover, FAP, the surrogate biomarker of CAF, was upregulated in relapsing tumor tissues but was downregulated in responders (38). Notably, the signatures of cytotoxic T cell and TGF-β signaling could also effectively predict treatment response to atezolizumab (38). In addition, in the single-institutional phase 2 trial NCT02658019 for advanced hepatocellular carcinoma (HCC), patients with low plasma TGF-β (< 200 pg/ml) at baseline had improved OS and PFS after anti-PD-1 treatment (39). Also, in non-small cell lung cancer (NSCLC), TGF-β concentration in the plasma collected seven days after anti-PD-1 treatment effectively predicted patient outcomes (133).

Transcriptomic data of microsatellite instability-high/mismatch repair-deficient gastrointestinal tumors showed TGF-β, EMT, Wnt/β-catenin, angiogenesis, hypoxia, KRAS, mTORC1, and metabolism-associated pathways were enriched in non-responders after PD-1 treatment (40). Similarly, the transcriptomic profile of metastatic bone and soft tissue sarcomas demonstrated that TGF-β signaling enrichment was negatively correlated with the efficacy of anti-PD-1 (134). Furthermore, the TGF-β signature (based on mRNA levels of *BMPR2*, *FKBP1A*, *SLC20A1*, *SKIL*, *TGFBR1*, and *XIAP*) predicted anti-PD-1/PD-L1 resistance in gynecologic cancer (135). The high TGF-β score was associated with shorter progression-free survival after immunotherapy (8.1 vs. 2.8 months, *P* < 0.05) (135). Additionally, for triple-negative breast cancer receiving Durvalumab with Nab-Paclitaxel, RNA-seq data showed that EMT, TGF-β, and extracellular matrix pathways were enriched in patients with residual disease (136).

## 6 TGF-β blockade enhancing the efficacy of anti-PD-1/PD-L1 therapy

Given the negative role of TGF-β signaling in cancer immunology and immunotherapy, it is rational to enhance ICI efficacy by blocking TGF-β. In preclinical explorations and clinical practice, combination therapies of TGF-β inhibitor and anti-PD-1/PD-L1, as well as anti-PD-L1/TGF-β bispecific antibodies/fusion proteins, have made rapid progress (137).

### 6.1 TGF-β inhibitor combined with anti-PD-1/PD-L1

The synergistic effect between TGF-β inhibitor (e.g. anti-TGF-β, receptor kinase inhibitor, cancer vaccine) and anti-PD-1/PD-L1 has been validated in multiple murine tumor models, including but not limited to CT26 (mouse colon cancer), MC38 (mouse colon cancer), 3LL (mouse Lewis lung cancer), and EMT-6 (mouse breast cancer) (47, 54, 138, 139). Mechanistically, the combination therapy reverses TGF-β-mediated immune exclusion, enhances immune infiltration, improves the activities of effectors, and alters the polarization of macrophages (140).

In the advanced NSCLC patients, the interim results of NCT03732274 showed that galunisertib (TGFβRI kinase inhibitor) combined with durvalumab (anti-PD-L1) had potent antitumor activity with a manageable safety profile (response rate: 30.8% for PD-L1≥1% tumors; response rate: 40.0% for PDL1≥25%) (141). However, in the single-arm, multicenter, phase Ib study NCT02734160, galunisertib plus durvalumab was tolerable in metastatic pancreatic cancer, in spite of the limited antitumor activity (142).

### 6.2 Anti-PD-L1/TGF-β bispecific antibody or bi-functional protein

Actually, most PD-1/PD-L1 and TGF-β dual blockade strategies in clinical practice are fulfilled by anti-PD-L1/TGF-β bispecific antibody or bi-functional protein, which has strategic advantages over the conventional two-agent combination. More importantly, due to the unique structure, bispecific antibodies or bi-functional proteins might have better tumor specificity and therapeutic effects (54, 82, 143). M7824 (fusion protein containing anti-PD-L1 and TGF-β trap) outperformed anti-PD-L1 and TGF-β trap in preclinical studies by mobilizing antitumor immunity (82, 144). Notably, in the phase 1 study NCT02517398, the response rate in NSCLC patients with high PD-L1 expression was high as 85.7% (83). Besides, the results of other early-stage clinical trials were encouraging as well (145). At present, the efficacy of M7824 is under evaluation in more than ten types of cancers, including NSCLC, triple-negative breast cancer, urothelial carcinoma, biliary tract cancer, gastric cancer, HPV-associated malignancies, and thymic carcinoma. Similarly, SHR-1701 (fusion protein of anti-PD-L1 antibody and TGF-β trap) exhibited encouraging antitumor activity in advanced tumors in the phase 1 study NCT03710265 (response rate: 17.8%) (146). Moreover, multiple phase 1/2 studies demonstrated the powerful antitumor activity of SHR-1701 in cervical cancer, *EGFR*-mutated NSCLC, biliary tract cancer, and pancreatic cancer (147–150)

YM101 is the first publicly reported anti-PD-L1/TGF-β bispecific antibody in the world (54). In the preclinical studies, YM101 overcame anti-PD-L1 resistance in 3LL, CT26, and EMT-6 tumor models (54). Investigations in the TME showed that YM101 expanded the numbers of TIL, M1-like macrophage, and DC, but decreased M2-like macrophage (54). The surrogate of YM101, Y101D is under evaluation in advanced solid tumors (NCT05028556).

## 7 Conclusions

TGF-β is a paradoxical regulator in cancer progression, which acts as a suppressor in early-stage cancer but as a promoter in advanced cancer. The negative effects of TGF-β on cancer immune surveillance have been well studied, including impairing immune infiltration, inducing the differentiation toward MDSC/M2-like macrophage/Treg, limiting the cytotoxicity of T cell and NK cell, and undermining the antigen presentation capability of DC. Accumulating evidence shows that TGF-β not only promotes cancer immune evasion but also predicts the efficacy of immune checkpoint inhibitors. Increased TGF-β level at baseline is commonly associated with a poor response to anti-PD-1/PD-L1 therapy. Blocking TGF-β could improve response to anti-PD-1/PD-L1 and patient outcomes. At present, dual PD-1/PD-L1 and TGF-β blockade have made a breakthrough, especially by anti-PD-L1/TGF-β bispecific antibody or bi-functional protein. This updated immune checkpoint inhibitor might alter the therapeutic paradigm for cancer in the future.

## Author contributions

MY and TL performed the selection of literature, drafted the manuscript and prepared the figures. MN and YW collected the related references and participated in discussion. KW and ZZ designed this review and revised the manuscript. All authors contributed to the article and approved the submitted version.
